# Structural Characterization of Withanolide Glycosides from the Roots of *Withania somnifera* and Their Potential Biological Activities

**DOI:** 10.3390/plants11060767

**Published:** 2022-03-13

**Authors:** Ji Won Ha, Jae Sik Yu, Bum Soo Lee, Dong-Min Kang, Mi-Jeong Ahn, Jung Kyu Kim, Ki Hyun Kim

**Affiliations:** 1 School of Pharmacy, Sungkyunkwan University, Suwon 16419, Korea; ellenha2@gmail.com (J.W.H.); jsyu@bu.edu (J.S.Y.); kosboybs@naver.com (B.S.L.); 2 New Material Development Team, COSMAX BIO Ltd., 255 Pangyo-ro, Bungdang-gu, Seongnam 13486, Korea; 3 College of Pharmacy and Research Institute of Pharmaceutical Sciences, Gyeongsang National University, Jinju 52828, Korea; kdm7105@gnu.ac.kr; 4 School of Chemical Engineering, Sungkyunkwan University, Suwon 16419, Korea

**Keywords:** *Withania somnifera*, withanolide glycosides, ECD, anti-*Helicobacter pylori* activity

## Abstract

*Withania somnifera* (Solanaceae), commonly known as “ashwagandha”, is an ayurvedic medicinal plant that has been used for promoting good health and longevity. As part of our ongoing natural product research for the discovery of bioactive phytochemicals with novel structures, we conducted a phytochemical analysis of *W. somnifera* root, commonly used as an herbal medicine part. The phytochemical investigation aided by liquid chromatography-mass spectrometry (LC/MS)-based analysis led to the isolation of four withanolide glycosides (**1**–**4**), including one new compound, withanoside XII (**1**), from the methanol (MeOH) extract of *W. somnifera* root. The structure of the new compound was determined by nuclear magnetic resonance (NMR) spectroscopic data, high-resolution (HR) electrospray ionization (ESI) mass spectroscopy (MS), and electronic circular dichroism (ECD) data as well as enzymatic hydrolysis followed by LC/MS analysis. In addition, enzymatic hydrolysis of **1** afforded an aglycone (**1a**) of **1**, which was identified as a new compound, withanoside XIIa (**1a**), by the interpretation of NMR spectroscopic data, HR-ESIMS, and ECD data. To the best of our knowledge, the structure of compound **2** (withagenin A diglucoside) was previously proposed by HRMS and MS/MS spectral data, without NMR experiment, and the physical and spectroscopic data of withagenin A diglucoside (**2**) are reported in this study for the first time. All the isolated compounds were evaluated for their anti-*Helicobacter pylori*, anti-oxidant, and anti-inflammatory activities. In the anti-*Helicobacter pylori* activity assay, compound **2** showed weak anti-*H. pylori* activity with 7.8% inhibition. All the isolated compounds showed significant ABTS radical scavenging activity. However, all isolates failed to show inhibitory activity against nitric oxide (NO) production in lipopolysaccharide-stimulated RAW 264.7 macrophage cells. This study demonstrated the experimental support that the *W. somnifera* root is rich in withanolides, and it can be a valuable natural resource for bioactive withanolides.

## 1. Introduction

*Withania somnifera* (L.) Dunal, also well-known as “Indian ginseng” or “ashwagandha” belongs to the plant family Solanaceae [1,2]. Its roots and leaves have been largely used for purposes to promote longevity and good health in the ayurvedic system of traditional Indian medicine for over 3000 years [2]. Until present, the roots of *W. somnifera* have been widely consumed as a functional food due to the fact of therapeutic properties, consisting of invigoration, enhancement of cognitive ability, and stress release activities. The root extracts are also commonly consumed as a powder, liquid, tablet, and capsule due to the fact of its prominent health benefits.

Withanolides are the primary constituents for the medicinal effects of this plant. It is defined as a class of steroidal lactones based on an ergostane skeleton, where C-22 and C-26 are oxidized to make a six-membered lactone ring [3]. Previous studies have reported withanolides as a high-priority topic for pharmacological research. Numerous reports showed a vast array of biological properties such as anticancer, neuroprotective, anti-inflammatory, immunomodulatory, and antioxidant activities of *W. somnifera* extracts and withanolides [4,5,6,7,8]. To date, more than 40 withanolides, including novel structural variants, such as withanone and withaferin A, have been isolated from *W. somnifera* [9]. Moreover, some reports have also found *W. somnifera* containing withanolide glycosides or glycowithanolides with a β-d-glucopyranose with glycosidic linkage at C-3 or C-27 [10]. Previous research has shown promising therapeutic potential for these withanolide glycosides, similar to the withanolide scaffold alone [11,12,13]. Novel glycosidic derivatives, such as withanosides I–XI, have been isolated from *W. somnifera* with reports of anti-Alzheimer’s, anti-stress, and neuroprotective activity [11]. Withanolide glycosides, possessing antiviral activity, have also proven to serve as potential therapeutic agents against COVID-19 [12,13]. Thus, withanolide glycosides have exhibited promising biological properties that can contribute to the bioactive constituents of natural medicines.

As part of a continuing natural product research to discover bioactive phytochemicals with novel structures [14,15,16,17], we explored bioactive phytochemicals from a methanolic extract of *W. somnifera* roots [18,19,20], commonly used as an herbal medicine part. In our recent studies of *W. somnifera* roots, we reported bioactive withanolides, including new compounds, namely, withasilolides A–F [18] and withasomniferol D [20], some of which showed cytotoxicity against some human cancer cells and anti-adipogenic activity. Moreover, new phenylpropanoid esters, namely, withaninsams A and B, along with known phenolic compounds and alkaloids showing anti-inflammatory effects were identified in our recent study [19]. As an ongoing study for the discovery of new metabolites from *W. somnifera* roots, we focused on polar fraction to isolate withanolide glycosides in the present study, since withanolide glycosides have been relatively un-investigated from *W. somnifera*. The intensive chemical analysis of the MeOH extracts of *W. somnifera* roots aided by liquid chromatography-mass spectrometry (LC/MS)-based analysis led to the isolation of four withanolide glycosides (**1**–**4**), including one new compound, withanoside XII (**1**), from the *n*-BuOH-soluble fraction. Enzymatic hydrolysis of **1** afforded a new compound, withanoside XIIa (**1a**), an aglycone of **1**. Herein, we describe the separation and structural elucidation of compounds **1**–**4** and evaluation of their anti-*Helicobacter pylori*, anti-oxidant, and anti-inflammatory activities.

## 2. Results and Discussion

### 2.1. Isolation of Compounds ***1**–**4***

**Figure 1:**
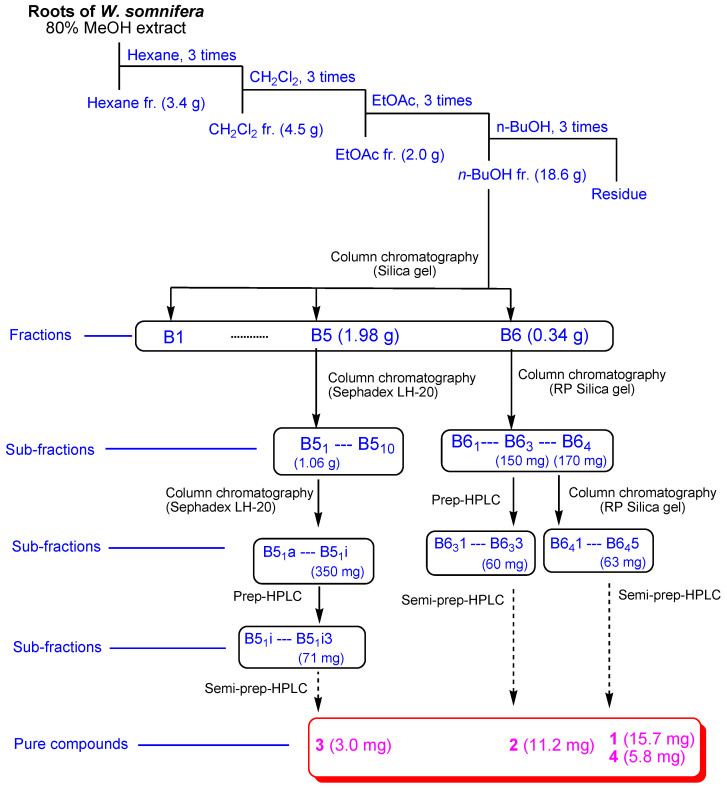
The separation scheme of compounds **1**–**4**.

**Figure 2:**
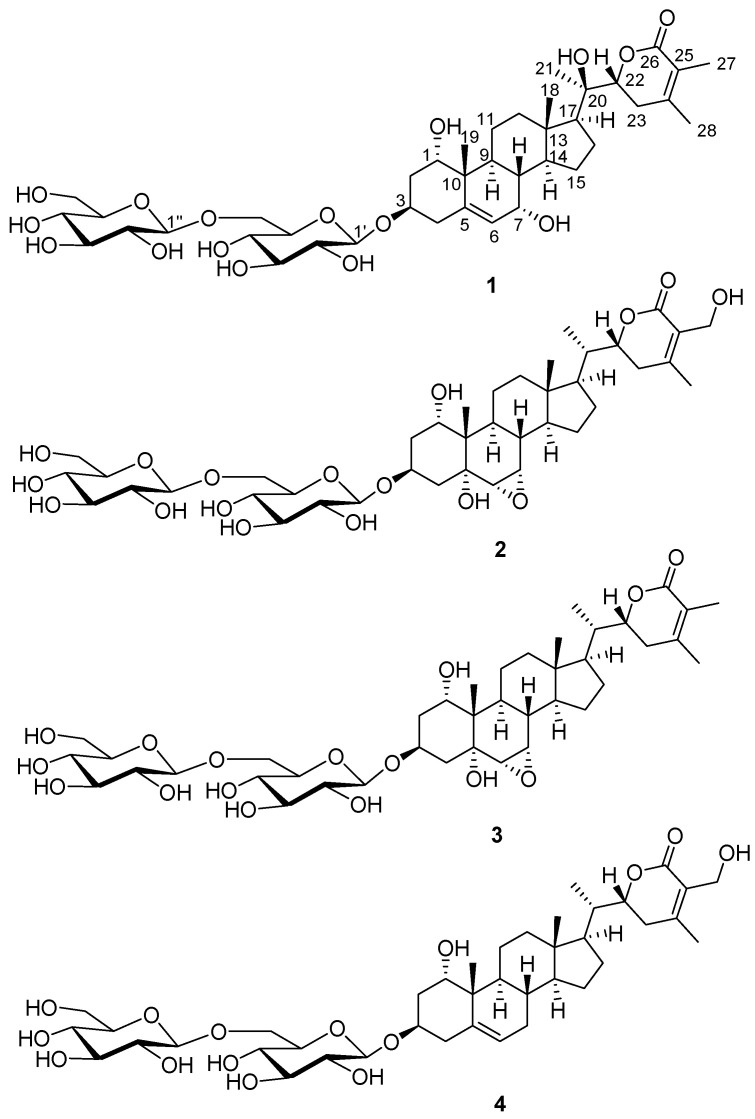
Chemical structures of compounds **1**–**4**.

The roots of *W. somnifera* were extracted using 80% MeOH under reflux to provide the crude MeOH extract followed by the rotary evaporation. The methanolic extract was sequentially applied to the solvent partition process by four solvents, namely, hexane, dichloromethane, ethyl acetate, and *n*-butanol, yielding four main solvent fractions (Figure 1). The LC/MS analysis of each fraction using reference to an in-house-built UV library database revealed that the *n*-butanol-soluble fraction contained withanolide glycosides. The phytochemical examination of the *n*-butanol fraction by application of repeated column chromatography and semi-preparative high-performance liquid chromatography (HPLC) (Figure 1) with the guidance of LC/MS analysis led to the separation of four withanolide glycosides (**1**–**4**) (Figure 2).

### 2.2. Structural Elucidation of the Isolated Compounds ***1**–**4***

Compound **1**, obtained as a white amorphous powder, possessed the molecular formula of C_40_H_62_O_16_ deduced from positive high-resolution electrospray ionization mass spectroscopy (HR-ESIMS), which revealed a [M + Na]^+^ ion peak at *m*/*z* 821.3920 (calcd. for C_40_H_62_ NaO_16_, 821.3936). The infrared (IR) spectrum of **1** showed distinctive absorptions for the hydroxy (3439 cm^−1^) and *α,β*-unsaturated ketone (1705 cm^−1^) functional groups. The ^1^H NMR data (Table 1) of compound **1**, assigned by the aid of heteronuclear single quantum correlation (HSQC) experiment, showed the presence of signals for five methyls (*δ*_H_ 0.89 (3H, s), 1.02 (3H, s), 1.29 (3H, s), 1.87 (3H, s), and 2.01 (3H, s)), four oxygenated methines (*δ*_H_ 3.75 (1H, t, *J* = 4.0 Hz), 3.86 (1H, br s), 4.09 (1H, m), and 4.27 (1H, dd, *J* = 13.5, 3.5 Hz)), and one olefinic proton (*δ*_H_ 5.77 (1H, d, *J* = 4.0 Hz)) as well as two indicative anomeric protons (*δ*_H_ 4.40 (1H, d, *J* = 8.0 Hz), 4.41 (1H, d, *J* = 8.0 Hz) for sugar moieties. The ^13^C NMR data (Table 1) of **1**, combined with heteronuclear multiple bond correlation (HMBC) experiment, revealed 40 carbon resonances, including 28 carbons for aglycone and 12 carbons for sugar units, where the carbon resonances assignable to the sugar units were typical of glucose [21]. Comprehensive inspection of the NMR data revealed that the NMR data of **1** was similar to those of withanoside VII, previously isolated from *W. somnifera* roots [22], but the apparent differences between the structures of **1** and withanoside VII were detected in the side chain due to the discrepancy of NMR data corresponding to C-20 and C-21. 

**Figure 3:**
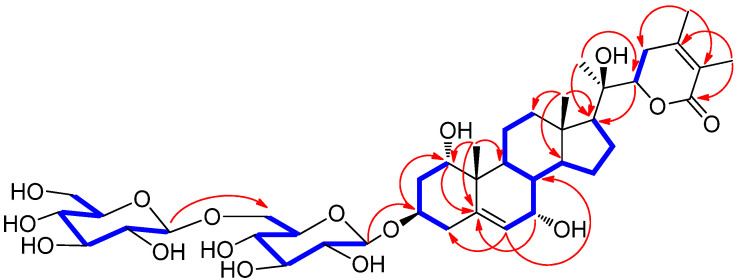
Key ^1^H-^1^H COSY (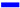) and HMBC (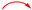) correlations for **1**.

The different partial structure of **1** was characterized by the analysis of the key HMBC correlations from H_3_-21 (*δ*_H_ 1.29)/C-17 (*δ*_C_ 54.3), C-20 (*δ*_C_ 74.9), and C-22 (*δ*_C_ 81.2) (Figure 3). Importantly, the linkage positions of the two glucoses were confirmed by the key COSY correlations from H-1 to H_2_-4, and key HMBC correlations of H-1′/C-3, and H-1″/C-6′ (Figure 3). Finally, the complete planar structure of **1** was elucidated by analysis of COSY and HMBC experiments (Figure 3).

**Figure 4:**
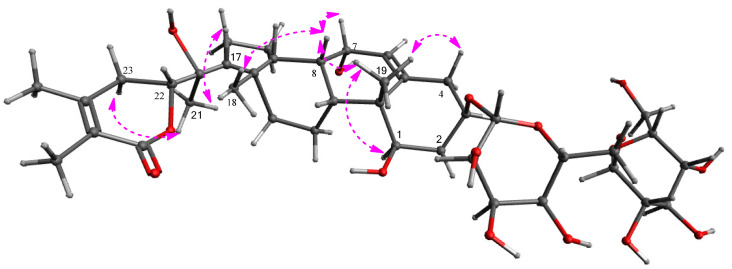
Key ROESY correlations for **1**.

The stereochemistry of **1** was established by the correlations obtained from the rotating frame Overhauser effect spectroscopy (ROESY) experiment, vicinal proton coupling constants observed in the ^1^H NMR specrtum, and electronic circular dichroism (ECD) data. The *α*-position of the hydroxy groups at C-1 and C-7 was determined by the ROESY correlations of H_3_-19/H-1 and H-4*β*, and H-8/H-7, H_3_-18, and H_3_-19 (Figure 4). The ECD spectrum of **1** showed a positive Cotton effect at 255 nm oriented from the n → π* transition of the *α*,*β*-unsaturated *δ*-lactone [18,23], which unambiguously confirmed a 22*R*-configuration. The configuration of C-22 was also supported by the typical coupling constants (*J* = 13.5 and 3.5 Hz) of H-22 showing a doublet of doublets [18,23]. In addition, almost the same ^13^C NMR chemical shifts observed for C-20 and the carbons of D ring and lactone ring of **1** to the related known withanolides, including withasilolide A [18], 20*β*-hydroxy-1-oxo-(22*R*)-witha-2,5,24-trienolide [24], withacoagin, [25], dunawithanine B [26], withacoagulin E [27], and 1*α*,3*β*,20*α*_F_-trihydroxy-20*R*,22*R*-witha-5,24-dienolide [28]. Suggested that the configuration of C-20 of **1** is the same as that of the related compounds. Finally, to identify the absolute configuration of sugar units, enzymatic hydrolysis of **1** using glucosidase was carried out, which afforded aglycone **1a** and the sugar moieties from **1**. The absolute configuration of two glucoses of **1** was concluded as D-configuration by LC/MS-based analysis after their thiocarbamoyl–thiazolidine derivatization [29]. The typical coupling constant (*J* = 8.0 Hz) of the anomeric protons was characteristic of *β*-form in glucopyranose [30], which demonstrated that both the sugar units of **1** were *β*-d-glucopyranoses. Therefore, the chemical structure of **1** was elucidated as shown in Figure 1, and compound **1** was named withanoside XII.

**Figure 5:**
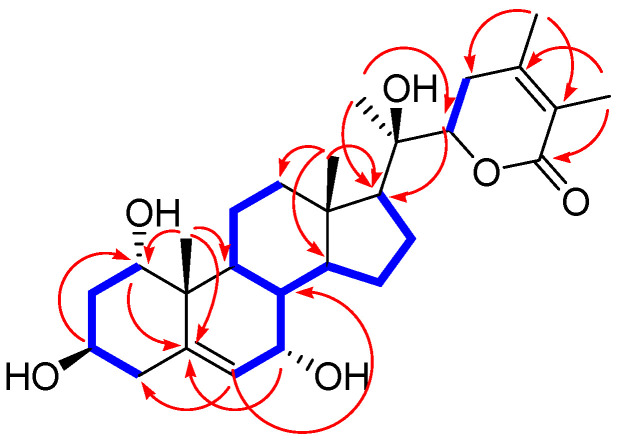
Key ^1^H-^1^H COSY (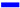) and HMBC (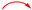) correlations for **1a**.

Compound **1a**, obtained as an aglycone of **1** by enzymatic hydrolysis, possessed the molecular formula of C_28_H_42_O_6_ confirmed by HRESIMS, which showed molecular ion peak at *m*/*z* 473.2904 [M − H]^−^ (calcd. for C_28_H_41_O_6_, 473.2903) in the negative-ion mode. The NMR data of **1a** showed clearly similar signals to those of compound **1**, without signals responsible for the sugar moieties. The up-field shifted signals (*δ*_H_ 3.91/*δ*_C_ 65.1) for C-3 of **1a** indicated the absence of the sugar units at C-3. Likewise, the planar structure of **1a** was further confirmed by the interpretation of COSY and HMBC experiments (Figure 5). The observed ROESY correlations and a positive Cotton effect at 251 nm in the ECD spectrum of **1a** confirmed the same stereochemistry of **1a** to that of **1**. Accordingly, the structure of **1a** was also assigned as a new compound, as illustrated in Figure 5 and compound **1a**, was named withanoside XIIa.

The known compounds were identified as withagenin A diglucoside (**2**) [31], withanoside II (**3**) [22], and withanoside IV (**4**) [22] by comparing their physical features and NMR spectroscopic data with those previously reported, and the data from LC/MS analysis. To the best of our knowledge, the compound **2** (withagenin A diglucoside) has not been isolated as a natural product, and the structure of withagenin A diglucoside (**2**) was previously proposed by HRMS and MS/MS spectral data without NMR experiment [31]. Compound **2** was isolated as a white amorphous powder, and the molecular formula of **2** was deduced as C_40_H_62_O_17_ from the (−)–HRESIMS data showing a [M − H]^−^ ion peak at *m*/*z* 813.3929 (calcd. for C_40_H_61_O_17_, 813.3909). Detailed ^1^H NMR data analysis of **2** revealed that the ^1^H NMR data of **2** was almost identical to those of withanoside II (**3**) [22], isolated in this study, with an apparent difference of one hydroxylated methylene group [*δ*_H_ 4.34 (1H, d, *J* = 12.0 Hz, 27-Ha) and 4.40 (1H, d, *J* = 12.0 Hz, 27-Hb)] of C-27 in **1**. Based on the NMR data, and the molecular formula (C_40_H_62_O_17_) determined by (−)–HRESIMS data, the compound **2** was unambiguously confirmed as withagenin A diglucoside, which was also supported by the comparable UV and ECD data of **2** to withanoside II (**3**) [22]. Here, we reported the physical and spectroscopic data of withagenin A diglucoside (**2**) for the first time.

### 2.3. Evaluation of Biological Activity of the Isolated Compounds

*Helicobacter pylori* is a major health problem worldwide, affecting approximately 50% of the global population [32,33]. In our anti-*H. pylori* activity assay, we found that the crude MeOH extract of *W. somnifera* roots showed weak antibacterial activity with 22.4% inhibition against *H. pylori* strain 51. Several previous studies have reported anti-bacterial activity of *W. somnifera*, and withaferin A, a major withanolide from this plant is known to inhibit *H. pylori*-induced inflammation [34,35,36,37]. Thus, the isolated withanolide glycosides (**1**–**4**) were evaluated for anti-*H. pylori* activity. Among the isolates, compound **2** exhibited weak anti-*H. pylori* activity with 7.8% growth inhibition against *H. pylori* strain 51, and the other compounds failed to show inhibitory activity. An epoxide group at C-6/C-7 and the hydroxyl groups at C-5 and C-27 may play a role in anti-*H. pylori* activity of compound **2**, compared with the structures of inactive compounds.

Anti-oxidant activity has been known to be related to various human diseases including cancer and inflammation, and the methanolic extract of *W. somnifera* has been reported to show anti-oxidant activity [36]. In this study, the crude MeOH extract of *W. somnifera* roots showed significant ABTS radical scavenging activity with 24.3 ± 4.9 μmol Trolox equivalent (TE)/g·DW (dry weight). Thus, the isolated compounds **1**–**4** were evaluated for anti-oxidant activity. Although the activity of **1**–**4** was lower than the extract, all the compounds also displayed significant anti-oxidant activity, and the TE values of **1**–**4** were 10.5 ± 3.2, 13.1 ± 2.1, 9.4 ± 1.8, and 11.6 ± 3.9 μmol TE/g·DW, respectively. Significant differences in the activity were not found among the isolated compounds.

In addition, the anti-inflammatory activity of the isolates was evaluated because ethanolic extract of *W. somnifera* root, withaferin A, and phenolic compounds from this plant have been reported to have anti-inflammatory activity [19,38]. However, all the isolates (**1**–**4**) failed to show inhibitory activity against lipopolysaccharide-induced nitric oxide (NO) production in RAW 264.7 murine macrophage cells.

## 3. Materials and Methods

### 3.1. General Experimental Procedure and Plant Material

Detailed information on the general experimental procedure and identification of plant material are provided in the Supplementary Materials.

### 3.2. Extraction and Separation of the Compounds

Dried roots of *W. somnifera* (1.28 kg) were extracted using 80% aqueous MeOH (each 3.0 L for 3 days) under reflux three times and filtered at room temperature. After the filtrate was concentrated through a rotavapor, the methanolic extract (189.6 g) was suspended in water (700 mL) and then partitioned with each 700 mL of hexane, dichloromethane (CH_2_Cl_2_), ethyl acetate (EtOAc), and *n*-butanol (*n*-BuOH). Four fractions were provided in corresponding order: hexane (3.4 g), CH_2_Cl_2_ (4.5 g), EtOAc (2.0 g), and *n*-BuOH soluble (18.6 g) fractions. Four fractions from the solvent partitioning were subjected to LC/MS analysis combined with the reference of an in-house-built UV spectra library, which indicated the presence of withanolide glycosides in the *n*-BuOH fraction since some peaks in the *n*-BuOH fraction exhibited the UV pattern (λ_max_ 200–230 nm) related to that reported for withanolides [18] and the molecular ion peaks ranging *m*/*z* 780–820.

The *n*-BuOH soluble fraction (18.6 g) was applied to silica gel column chromatography (150 g, eluted with CH_2_Cl_2_/MeOH (30:1 → 1:1) to give six fractions (B1–B6)). Fraction B5 (1.98 g) was subjected to Sephadex LH-20 column chromatography (100% MeOH) to yield ten subfractions (B5_1_–B5_10_). Subfraction B5_1_ (1.06 g) was separated by Sephadex LH-20 column chromatography again to yield nine subfractions (B5_1_a–B5_1_i). Subfraction B5_1_i (350 mg) was further separated through preparative HPLC (65% MeOH → 80% MeOH, gradient solvent system) to give three subfractions (B5_1_i1–B5_1_i3). Subfraction B5_1_i3 (71 mg) was purified using semi-preparative HPLC (66% MeOH) to yield compound **3** (*t*_R_ 35.3 min, 3.0 mg). Fraction B6 (340 mg) was subjected to reverse-phase (RP) silica gel column chromatography (40% MeOH → 100% MeOH, gradient solvent system) twice, yielding 4 subfractions (B6_1_–B6_4_), and subfraction B6_3_ (150 mg) was applied to preparative HPLC (65% MeOH → 80% MeOH, gradient solvent system) to give three subfractions (B6_3_1–B6_3_3). Subfraction B6_3_3 (60 mg) was separated using semi-preparative HPLC (49% MeOH) to give compound **2** (*t*_R_ 60.0, 11.2 mg). Subfraction B6_4_ (170 mg) was fractionated by RP silica gel column chromatography (40% MeOH → 100% MeOH, gradient solvent system) to yield five subfractions (B6_4_1–B6_4_5). Finally, subfraction B6_4_5 (63 mg) was purified by semi-preparative HPLC (53% MeOH) to give compounds **4** (*t*_R_ 30.0, 5.8 mg) and **1** (*t*_R_ 34.8, 15.7 mg).

#### 3.2.1. Withanoside XII (**1**)

White amorphous powder; ${\left(\alpha \right)}_{D}^{25}\;$−24.9 (*c* 0.78, MeOH); UV (MeOH) λ_max_ (log ε) 194 (2.8) nm; IR (KBr) ν_max_ 3439, 2921, 1705, 1643, 1411, and 1034 cm^−1^; ECD (MeOH) λ_max_ (Δ*ε*) 221 (+0.37), 255 (+16.5) nm; ^1^H (850 MHz) and ^13^C NMR (212.5 MHz), see Table 1; (+)–ESIMS *m*/*z* 821 [M + Na]^+^; (+)–HRESIMS *m*/*z* 821.3920 [M + Na]^+^ (calcd. for C_40_H_62_ NaO_16_, 821.3936).

#### 3.2.2. Withagenin A Diglucoside (**2**)

White amorphous powder; ${\left(\alpha \right)}_{D}^{25}\;$−18.4 (*c* 0.55, MeOH); UV (MeOH) λ_max_ (log ε) 228 (3.8) nm; IR (KBr) ν_max_ 3435, 2931, 1698, 1645, 1387, and 1044 cm^−1^; ECD (MeOH) λ_max_ (*Δε*) 250 (+5.5) nm; ^1^H NMR (850 MHz CD_3_OD) *δ*: 0.85, 0.90, 2.13 (3H each, all s, 18, 19, 28-H_3_), 1.06 (3H, d, *J* = 7.0 Hz, 21-H_3_), 2.95 (1H, d, *J* = 4.0 Hz, 6-H), 3.25 (1H, dd-like, 7-H), 3.72 (1H, t, *J* = 3.0 Hz, 1-H), 4.34 (1H, d, *J* = 12.0 Hz, 27-Ha), 4.40 (1H, d, *J* = 12.0 Hz, 27-Hb), 4.43 (1H, d, *J* = 8.0 Hz, 1″-H), 4.45 (1H, d, *J* = 8.0 Hz, 1-H), 4.47 (1H, overlap, 3-H), and 4.50 (1H, m, 22-H); (−)–ESIMS *m*/*z* 813 [M − H]^−^. 

### 3.3. Enzymatic Hydrolysis and Absolute Configuration Determination of the Sugar Moieties of Compound ***1***

The absolute configurations of the sugar moieties were determined using the method previously described [24] with minor modifications. Briefly, compound **1** (1.0 mg) was hydrolyzed with crude glucosidase (10 mg, from almonds, Sigma-Aldrich, St. Louis, MO, USA) for 72 h at 37 °C, and CH_2_Cl_2_ was used for the extraction of aglycone. The CH_2_Cl_2_ fraction was confirmed to contain the aglycone **1a** by LC/MS analysis. Detailed description for absolute configuration determination of the sugar moieties in the aqueous layer is provided in the Supplementary Materials.

#### Withanoside XIIa (**1a**)

White amorphous powder; ${\left(\alpha \right)}_{D}^{25}\;$−46.5 (*c* 0.03, MeOH); UV (MeOH) *λ*_max_ (log ε) 200 (2.6) nm; IR (KBr) ν_max_ 3715, 2940, 2834, 1698, 1555, 1112, and 1025 cm^−1^; ECD (MeOH) λ_max_ (Δε) 219 (+8.5), 251 (+17.3) nm; ^1^H (850 MHz) and ^13^C NMR (212.5 MHz), see the Table 1; (−)–ESIMS *m*/*z* 519 [M + HCOOH]^−^; (−)–HRESIMS *m*/*z* 473.2904 [M − H]^−^ (calcd. for C_28_H_41_O_6_, 473.2903).

### 3.4. Anti-Helicobacter pylori Activity

The anti-*H. pylori* activity was evaluated using a previously described method [39]. The detailed information on the experimental procedure is included in the Supplementary Materials.

### 3.5. Antioxidant Activity Test with ABTS Radical

The ABTS radical was generated using a previously reported method [40]. Each sample (20 μL) was reacted with 180 μL of the ABTS^•+^ solution at room temperature, and the absorbance was measured at 734 nm after 10 min. The antioxidant activity of each sample was expressed as Trolox (Sigma, St. Louis, MO, USA) equivalents per gram (μmol TE/g).

### 3.6. Anti-Inflammatory Activity

The anti-inflammatory activity was evaluated using our previously reported method [19]. The detailed information on the experimental procedure is included in the Supplementary Materials.

### 3.7. Statistical Analysis

All of the contents and the antioxidant activities are expressed as the means ± standard deviations (SDs) of triplicate determinations. The differences among samples were statistically evaluated via one-way analysis of variance (ANOVA). The values were evaluated at the 5% significance level using two-sided tests. Pearson’s correlation coefficients were obtained using IBM SPSS Statistics 24.0 software (Armonk, NY, USA).

## 4. Conclusions

In conclusion, we isolated and characterized four withanolide glycosides (**1**–**4**), including one new compound, withanoside XII (**1**), in the *n*-BuOH fraction of the MeOH extracts of *W. somnifera* root via the LC/MS-based analysis [41]. The structure of withanoside XII was established by 1D and 2D NMR spectroscopic methods, HR-ESIMS, and ECD data as well as enzymatic hydrolysis followed by LC/MS analysis. We also characterized the new compound, withanoside XIIa (**1a**), as an aglycone of **1**, obtained by enzymatic hydrolysis of **1**. The physical and spectroscopic data of withagenin A diglucoside (**2**) were reported in this study for the first time. In the anti-*H. pylori* activity assay, compound **2** showed weak anti-*H. pylori* activity with 7.8% inhibition. In addition, all the isolated withanolide glycosides showed significant anti-oxidant activity in the ABTS radical scavenging assay. The present study provided the experimental support that *W. somnifera* root is rich in withanolides, and it can be a valuable natural resource for bioactive withanolides.

## Figures and Tables

**Table 1:** ^1^H (850 MHz) and ^13^C NMR (212.5 MHz) data of compounds **1** and **1a** in CD_3_OD (*δ* in ppm) ^a^.

Position	1		1a	
	*δ*_H_ (*J* in Hz)	*δ*_C_	*δ*_H_ (*J* in Hz)	*δ*_C_
1	3.86 dd (1.5, 1.5)	72.2 d	3.82 dd (1.0, 1.0)	72.0 d
2*α*	2.55 ddd (13.0, 5.5, 1.5)	37.7 t	2.01 ddd (13.0, 5.5, 1.0)	37.6 t
2*β*	2.40 ddd (13.0, 12.5, 1.5)		1.73 ddd (13.0, 12.5, 1.0)	
3	4.09 dddd (12.5, 12.5, 5.5, 5.5)	73.8 d	3.91 dddd (12.5, 12.5, 5.5, 5.5)	65.1 d
4*α*	2.23 dd (13.0, 5.5)	36.1 t	2.36 dd (13.0, 5.5)	40.9 t
4*β*	1.85 dd (13.0, 12.5)		2.32 dd (13.0, 12.5)	
5		142.7 s		142.9 s
6	5.77 d (4.0)	126.2 d	5.72 d (4.0)	126.0 d
7	3.75 t (4.0)	64.4 d	3.72 t (4.0)	64.5 d
8	1.44 m	37.0 d	1.46 m	37.0 d
9	1.92 m	33.4 d	1.92 m	33.8 d
10		41.8 s		41.6 s
11*α*	1.57 m	19.5 t	1.51 m	19.3 t
11*β*	1.52 m		1.56 m	
12*α*	1.37 m	39.2 t	2.00 m	39.3 t
12*β*	2.01 m		1.36 m	
13		42.3 s		42.1 s
14	1.57 m	49.6 d	1.57 m	49.8 d
15*α*	1.86 m	23.1 t	1.19 m	22.3 t
15*β*	1.22 m		1.84 m	
16*α*	1.70 m	21.5 t	1.69 m	21.3 t
16*β*	1.91 m		1.91 m	
17	1.82 m	54.3 d	1.82 m	54.1 d
18	0.89 s	12.6 q	0.89 s	12.6 q
19	1.02 s	17.5 q	1.02 s	17.3 q
20		74.9 s		75.0 s
21	1.29 s	19.5 q	1.28 s	19.3 q
22	4.27 dd (13.5, 3.5)	81.2 d	4.25 dd (13.5, 3.5)	81.0 d
23*α*	2.32 m	30.8 t	2.32 m	30.8 t
23*β*	2.56 m		2.54 t (15.0)	
24		151.5 s		151.2 s
25		120.6 s		120.6 s
26		167.8 s		167.6 s
27	1.87 s	11.1 q	1.87 s	10.8 q
28	2.01 s	19.2 q	2.00 s	19.1 q
1′	4.40 d (8.0)	101.8 d		
2′	3.18 dd (9.0, 8.0)	73.8 d		
3′	3.38 overlap	76.4 d		
4′	3.28 overlap	70.3 d		
5′	3.49 overlap	75.6 d		
6′	4.14 dd (12.0, 2.0); 3.80 dd (12.0, 6.0)	68.5 t		
1″	4.41 d (8.0)	103.5 d		
2″	3.23 (9.0, 8.0)	73.8 d		
3″	3.36 overlap	76.4 d		
4″	3.30 overlap	70.0 d		
5″	3.28 overlap	76.5 d		
6″	3.88 dd (12.0, 2.0); 3.69 dd (12.0, 5.5)	61.3 t		

^a^ *J* values are in parentheses (shown in Hz); ^13^C NMR assignments were based on HSQC and HMBC experiments.

## Data Availability

Not applicable.

## Supplementary Materials

The following supporting information can be downloaded at: https://www.mdpi.com/article/10.3390/plants11060767/s1, Figure S1: The HR-ESIMS data of **1**; Figure S2: The ^1^H NMR spectrum of **1** (CD_3_OD, 850 MHz); Figure S3: The ^1^H-^1^H COSY spectrum of **1** (CD_3_OD); Figure S4: The HSQC spectrum of **1** (CD_3_OD); Figure S5: The HMBC spectrum of **1** (CD_3_OD); Figure S6: The ROESY spectrum of **1** (CD_3_OD); Figure S7: The ECD spectrum of **1**; Figure S8: The HR-ESIMS data of **1a**; Figure S9: The ^1^H NMR spectrum of **1****a** (CD_3_OD, 850 MHz); Figure S10: The ^1^H-^1^H COSY spectrum of **1a** (CD_3_OD); Figure S11: The HSQC spectrum of **1a** (CD_3_OD); Figure S12: The HMBC spectrum of **1a** (CD_3_OD); Figure S13: The ROESY spectrum of **1a** (CD_3_OD); Figure S14: The ECD spectrum of **1a**; Figure S15: The HR-ESIMS data of **2**; Figure S16: The ^1^H NMR spectrum of **2** (CD_3_OD, 850 MHz); General experimental procedure.
