# Supplementary material for: Structural Characterization of Withanolide Glycosides from the Roots of Withania somnifera and Their Potential Biological Activities
Source: Plants (Basel). 2022 Mar 13;11(6):767. doi: 10.3390/plants11060767 (PMC8954635; doi:10.3390/plants11060767)
Supplement: Supplementary file 1 [file plants-11-00767-s001.zip › plants-1636380-supplementary.pdf]

Supplementary data

Structural Characterization of Withanolide Glycosides from the Roots of *Withania somnifera*

Ji Won Ha [1], Jae Sik Yu [1,2], Bum Soo Lee [1], Dong-Min Kang [3], Mi-Jeong Ahn [3], Jung Kyu Kim [4,*], and Ki Hyun Kim [1,*]

[1] School of Pharmacy, Sungkyunkwan University, Suwon 16419, Korea; ellenha2@gmail.com (J.W.H.); jsyu@bu.edu (J.S.Y); kosboybs@naver.com (B.S.L.)

[2] New Material Development Team, COSMAX BIO Ltd., 255 Pangyo-ro, Bungdang-gu, Seongnam 13486, Gyeonggi-do, Republic of Korea

[3] College of Pharmacy and Research Institute of Pharmaceutical Sciences, Gyeongsang National University, Jinju 52828, Korea; kdm7105@gnu.ac.kr (D.M.K.); amj5812@gnu.ac.kr (M.J.A.)

[4] School of Chemical Engineering, Sungkyunkwan University, Suwon, Gyeonggi-do, 16419, Republic of Korea

***** Correspondence: legkim@skku.edu (J.K.K.); khkim83@skku.edu (K.H.K.); Tel.: +82-31-290-7254 (J.K.K.); +82-31-290-7700 (K.H.K.)

Supporting Information Contents:

Figure S1. The HR-ESIMS data of **1**……………..…………………………………………………………………………………….S3
Figure S2. The ^{1}H NMR spectrum of **1** (CD_3OD, 850 MHz)...…..…………………………………………………………………….S4
Figure S3. The ^{1}H-^{1}H COSY spectrum of **1** (CD_3OD)..…………………………….……………………….……………………S5
Figure S4. The HSQC spectrum of **1** (CD_3OD).………………………………….…………………….……………………S6
Figure S5. The HMBC spectrum of **1** (CD_3OD)….………………………………………………………………………………S7
Figure S6. The ROESY spectrum of **1** (CD_3OD)…………………………………………………………………………………S8
Figure S7. The ECD spectrum of **1**…………………………………………………………………………………………… S9
Figure S8. The HR-ESIMS data of **1a**……………..…………………………………………………………………………………S10
Figure S9. The ^{1}H NMR spectrum of **1a** (CD_3OD, 850 MHz)...…..……………………………………………………………………….S11
Figure S10. The ^{1}H-^{1}H COSY spectrum of **1a** (CD_3OD)..……………………………….……………………….……………………S12
Figure S11. The HSQC spectrum of **1a** (CD_3OD).…………………………………….…………………….……………………S13
Figure S12. The HMBC spectrum of **1a** (CD_3OD)….……………………………………………………………………………S14
Figure S13. The ROESY spectrum of **1a** (CD_3OD)………………………………………………………………………………S15
Figure S14. The ECD spectrum of **1a**………………………………………………………………………………………….S16
Figure S15. The HR-ESIMS data of **2**…………………....…………………………………………………………………………S17
Figure S16. The ^{1}H NMR spectrum of **2** (CD_3OD, 850 MHz)...…..……………………….………………………………………….S18

General experimental procedure………………………………………………………………………………………….…….S19

Figure S1. The HR-ESIMS data of **1**

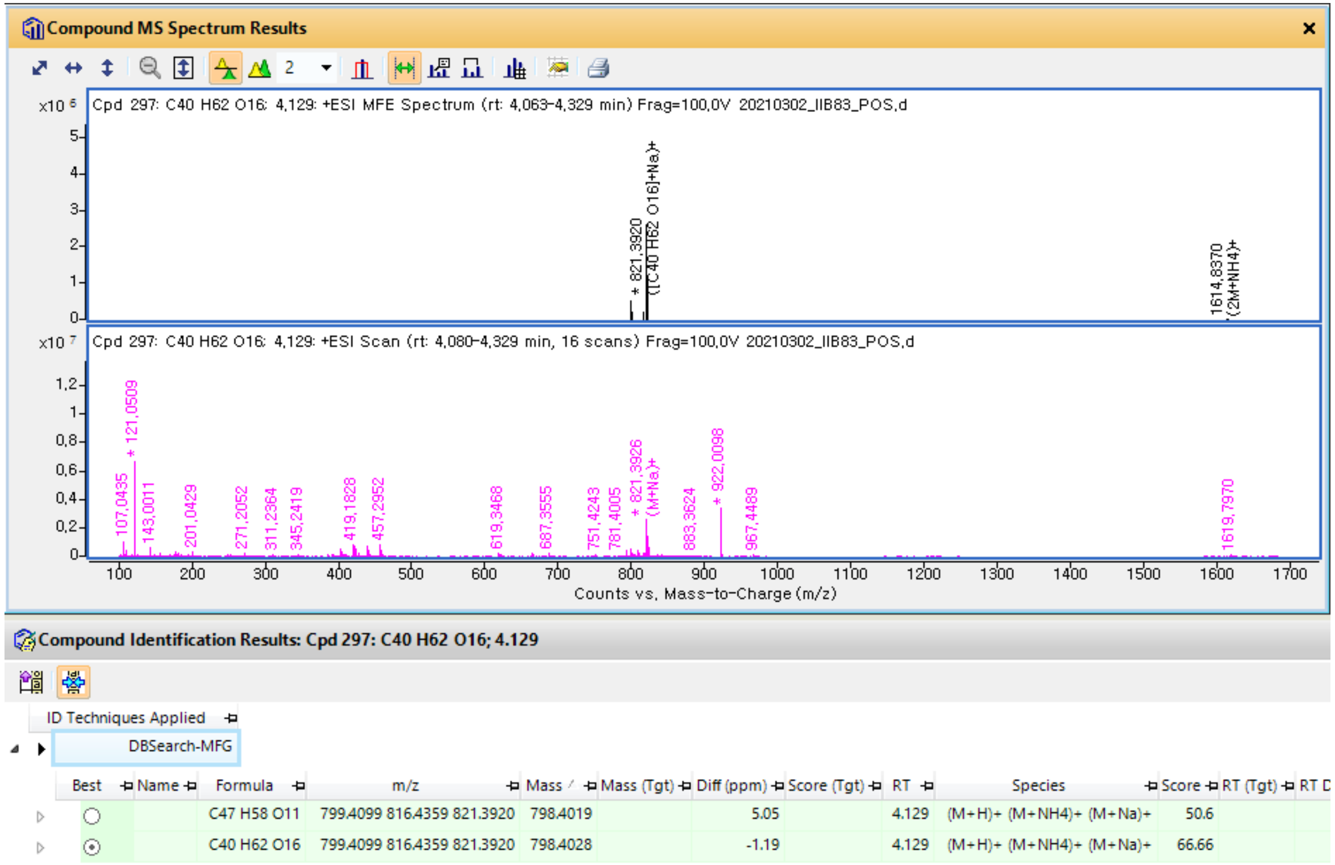

Figure S2. The ^{1}H NMR spectrum of **1** (CD_3OD, 850 MHz)

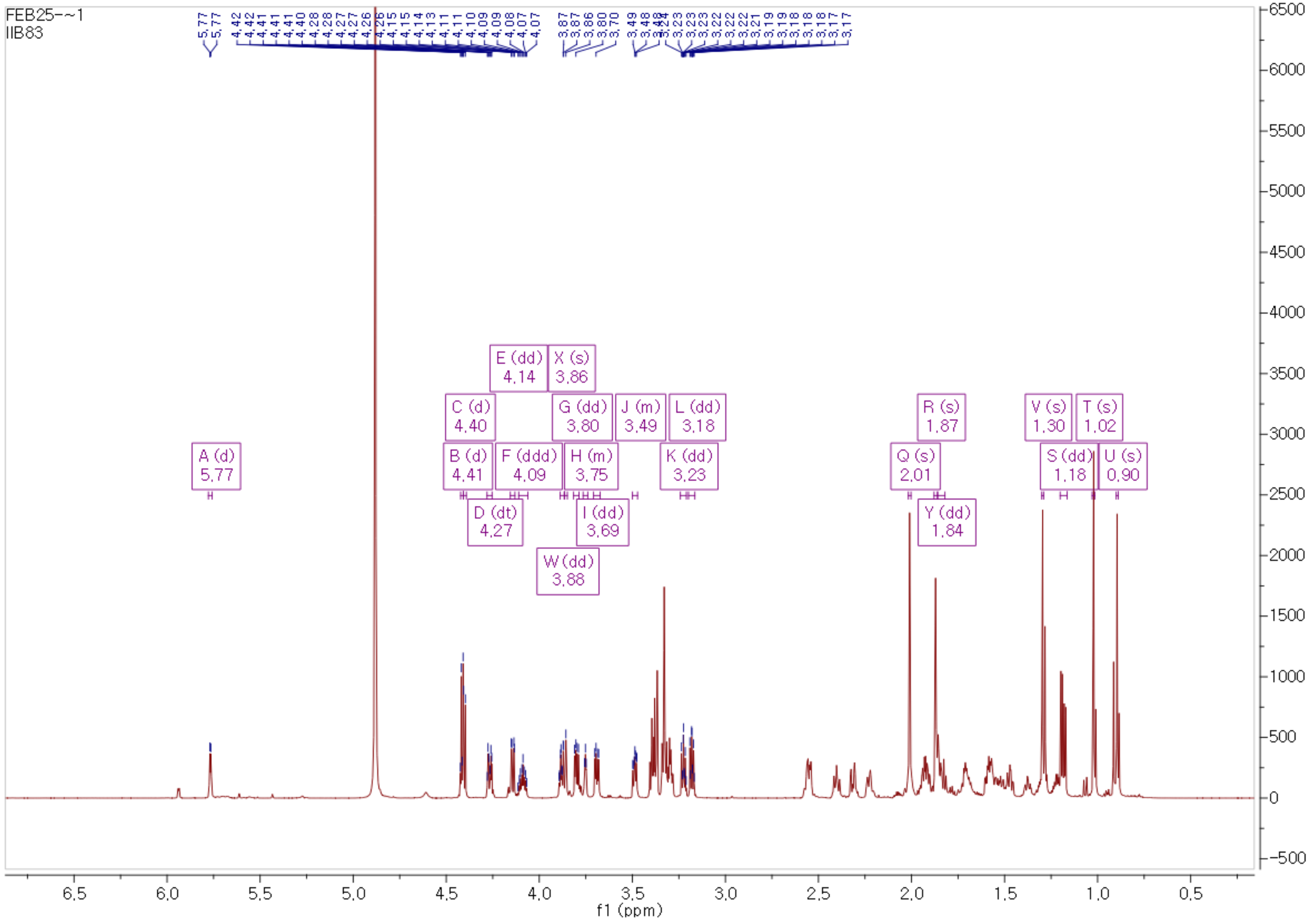

Figure S3. The ^{1}H-^{1}H COSY spectrum of **1** (CD_3OD)

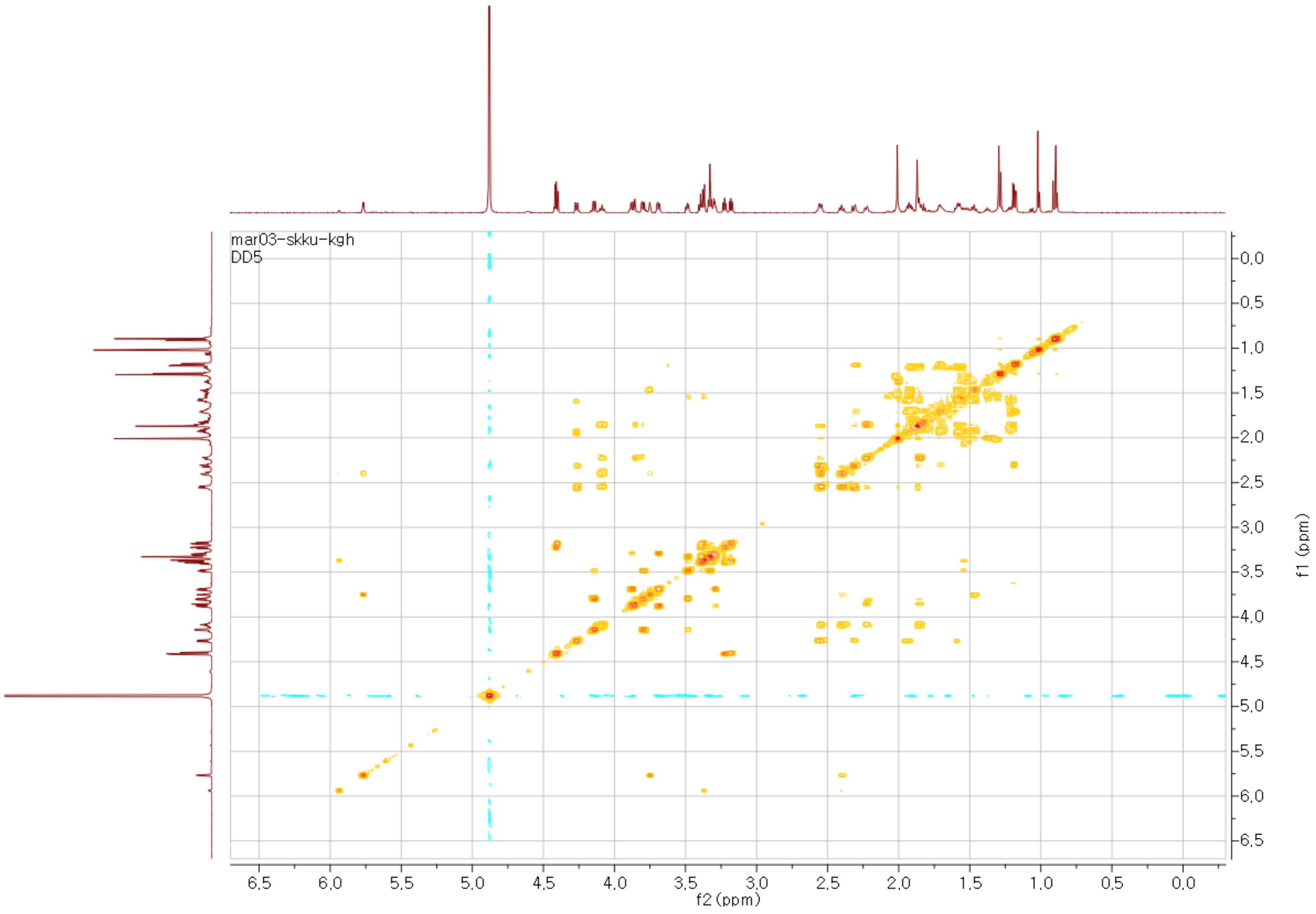

Figure S4. The HSQC spectrum of **1** (CD_3OD)

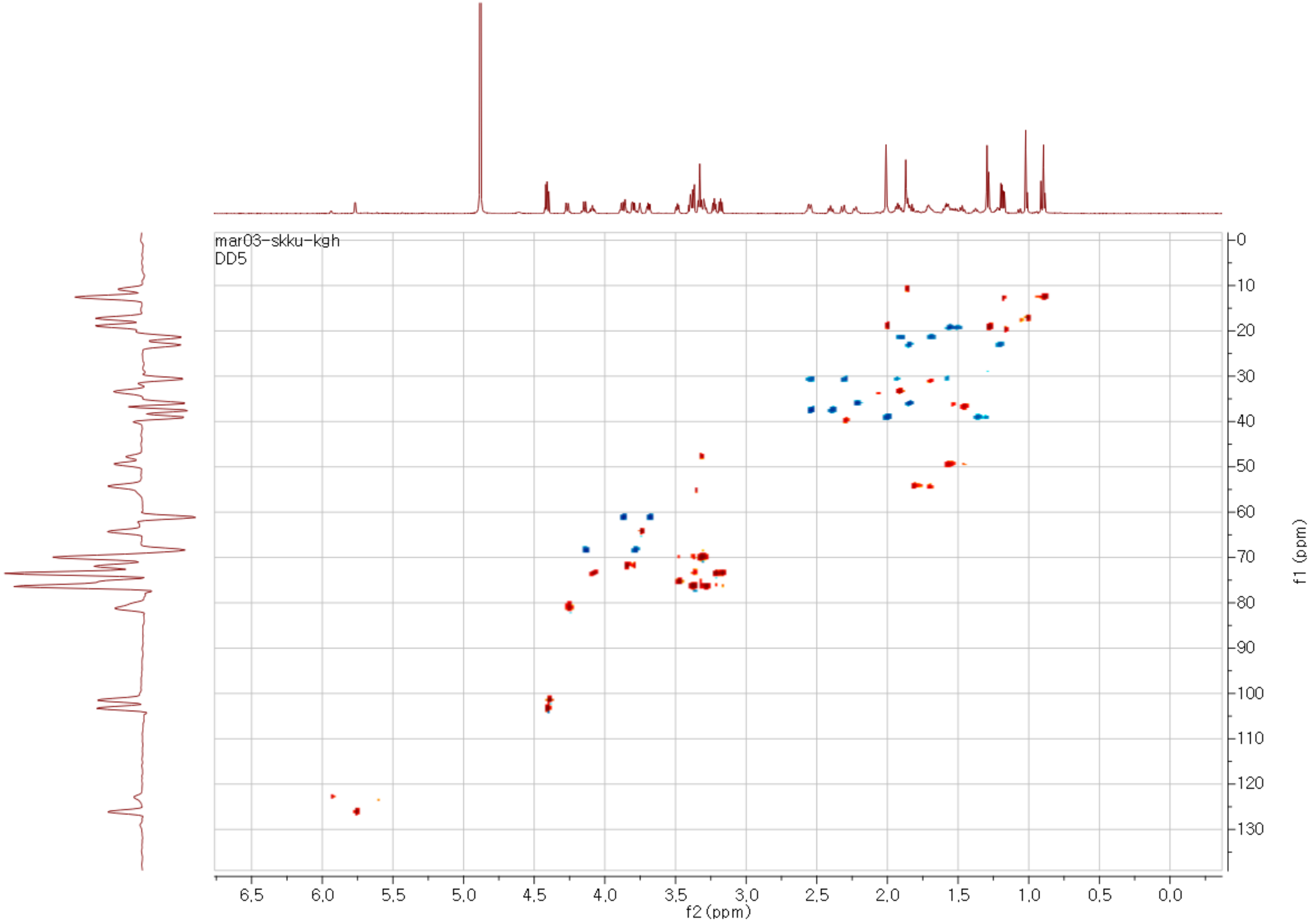

Figure S5. The HMBC spectrum of **1** (CD_3OD)

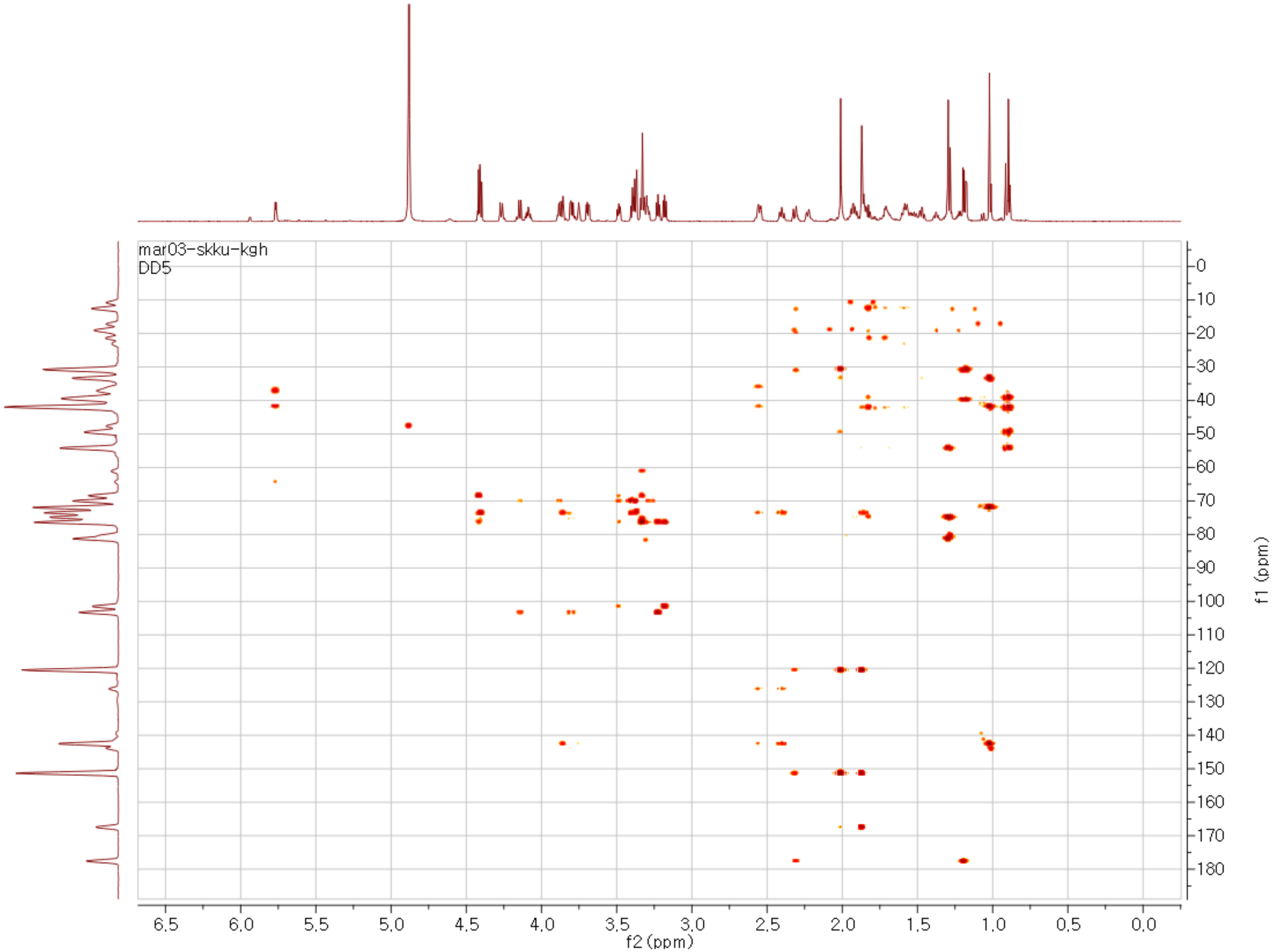

Figure S6. The ROESY spectrum of **1** (CD_3OD)

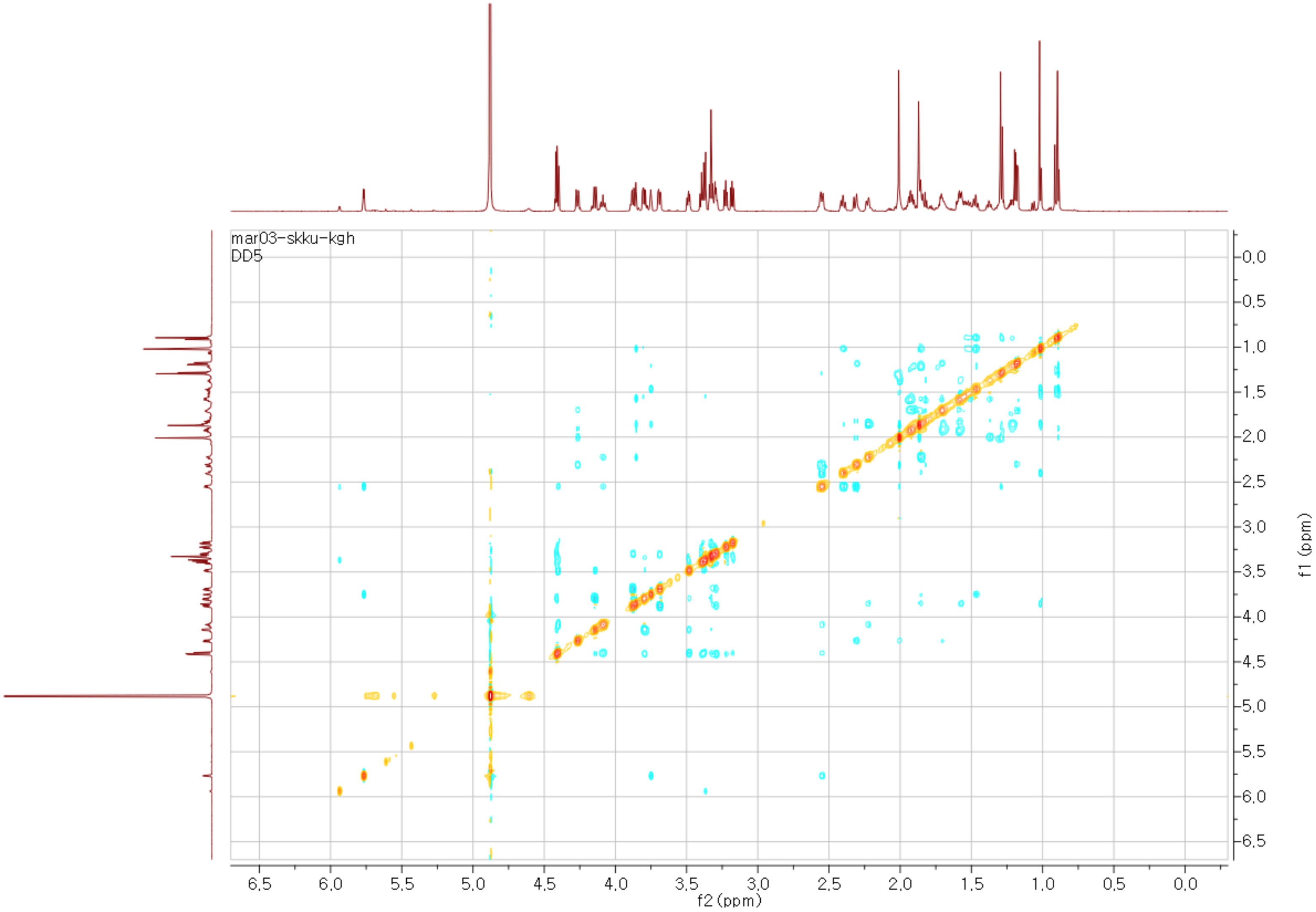

Figure S7. The ECD spectrum of **1**

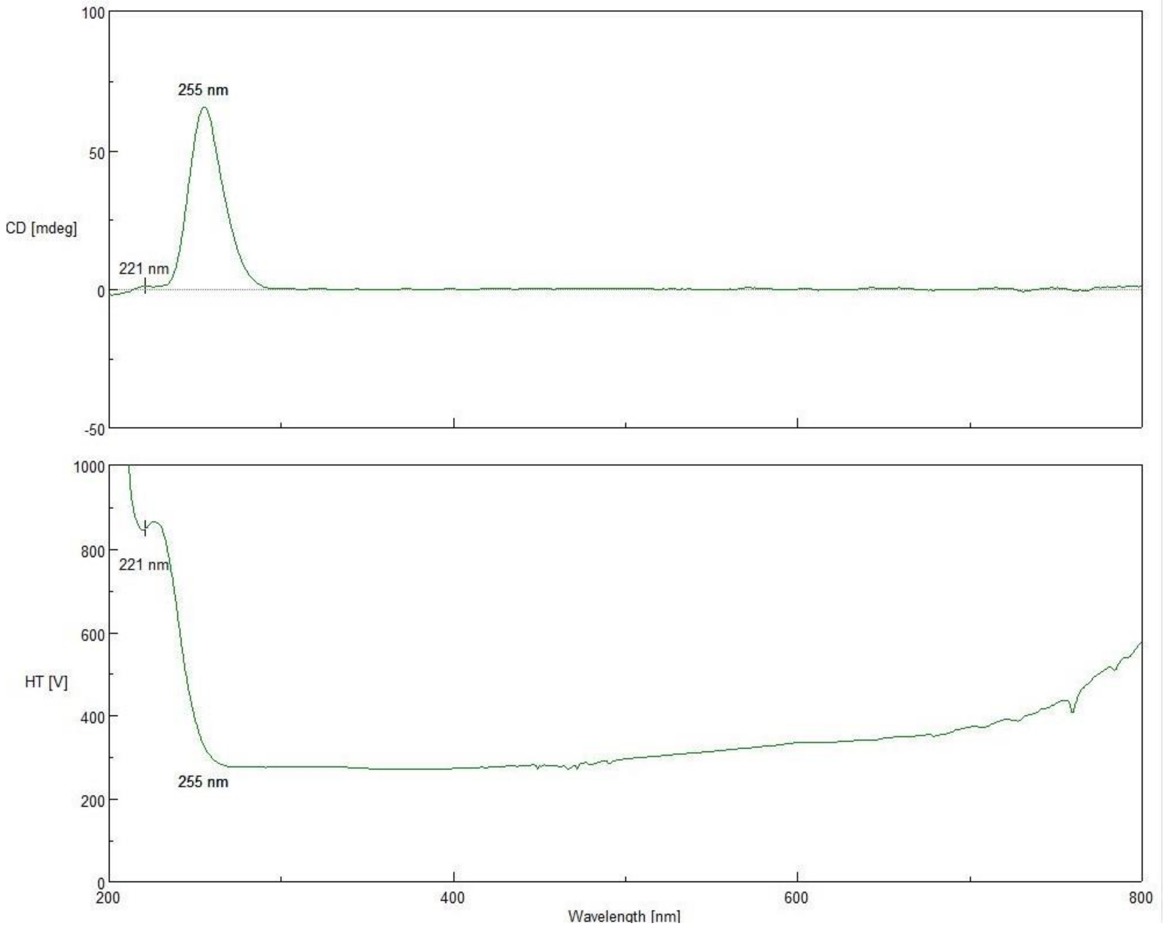

Figure S8. The HR-ESIMS data of **1a**

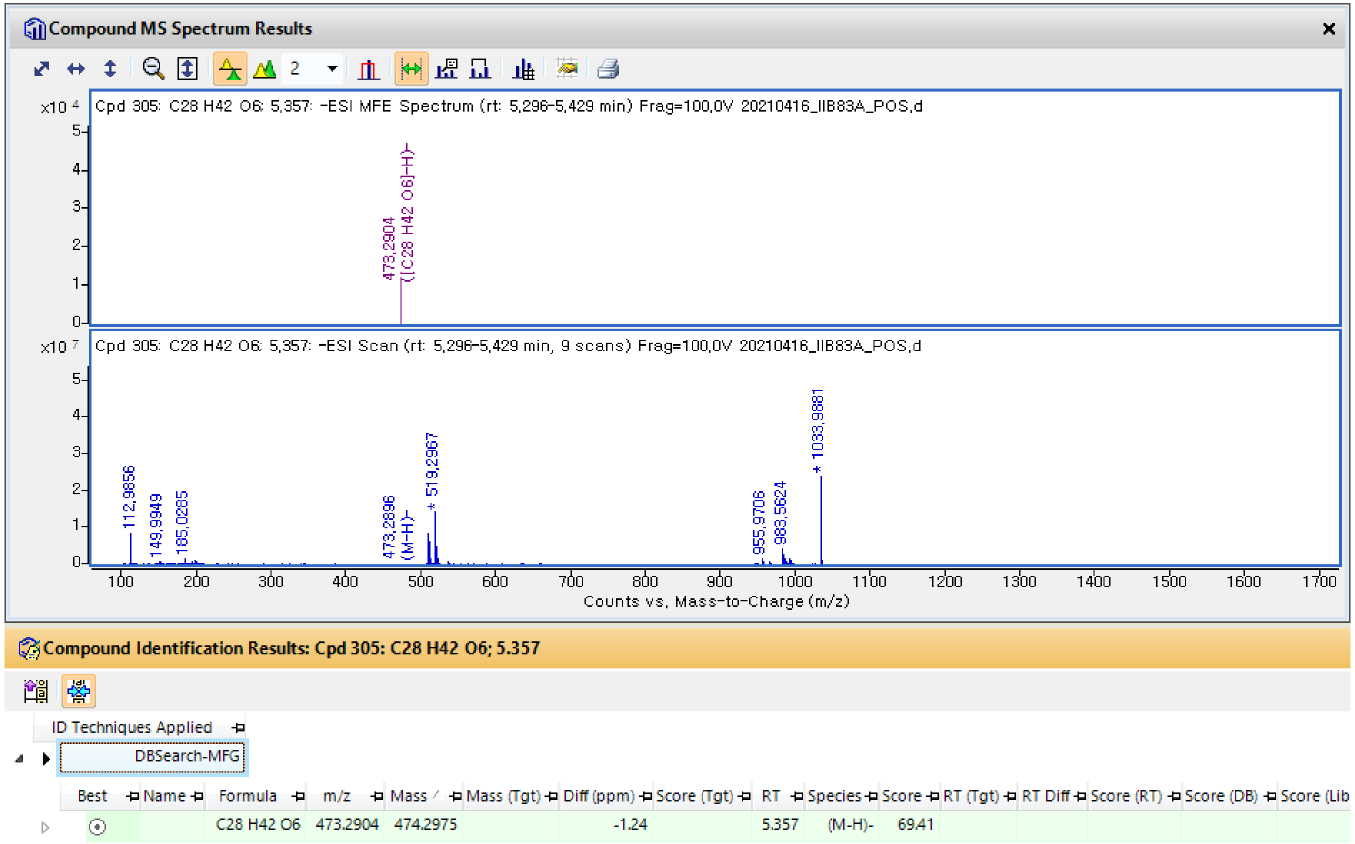

Figure S9. The ^{1}H NMR spectrum of **1a** (CD_3OD, 850 MHz)

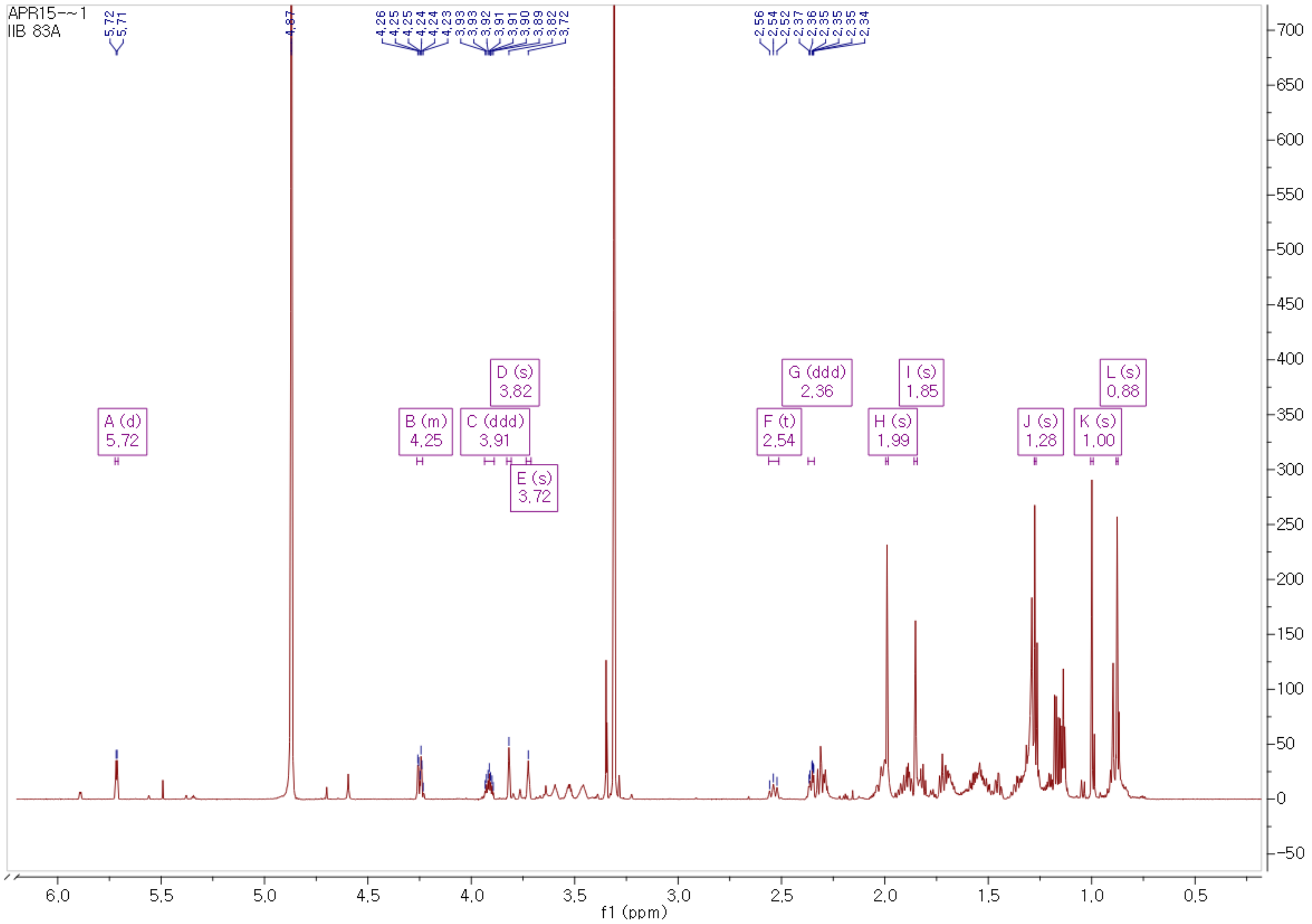

Figure S10. The ^{1}H-^{1}H COSY spectrum of **1a** (CD_3OD)

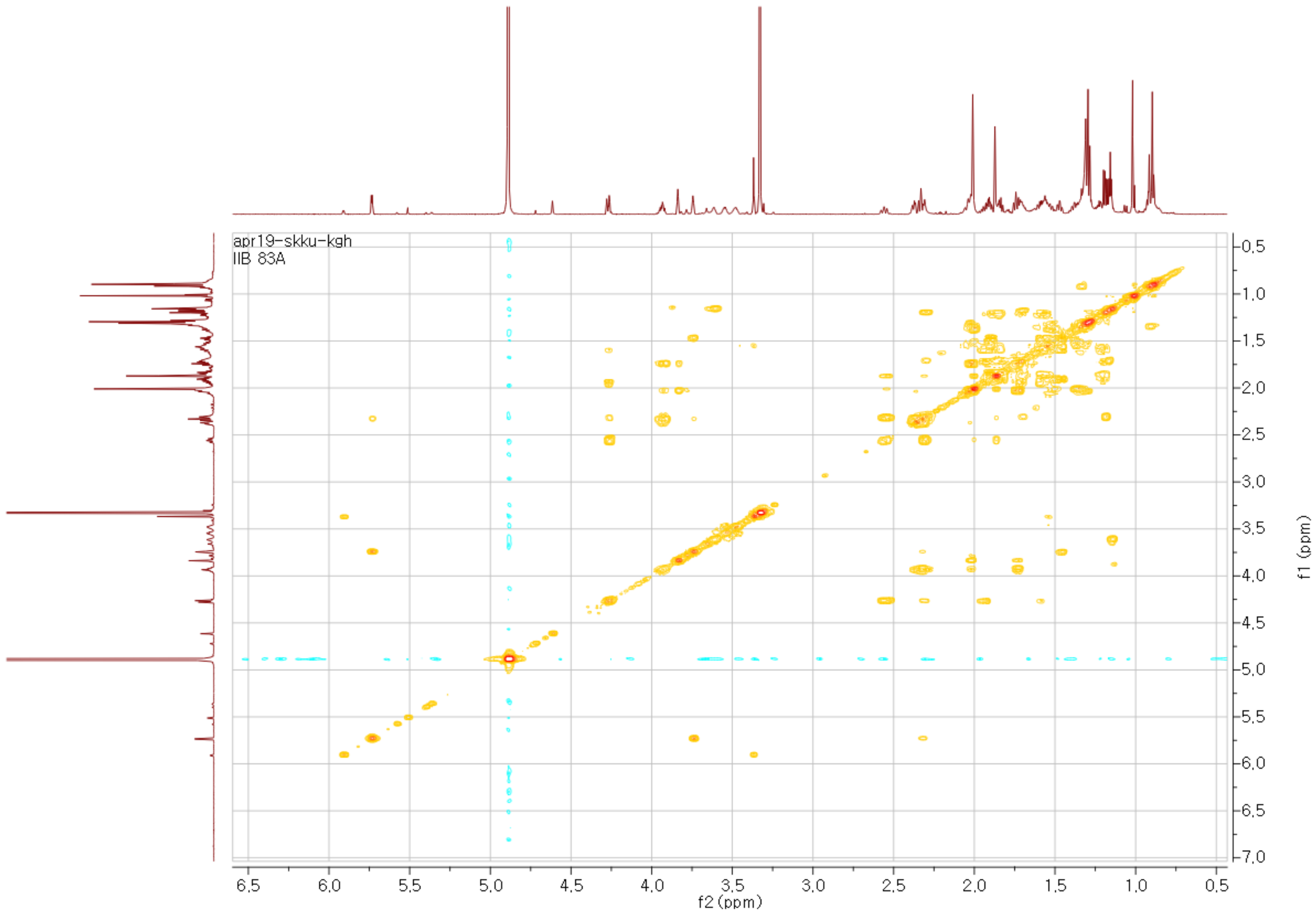

Figure S11. The HSQC spectrum of **1a** (CD_3OD)

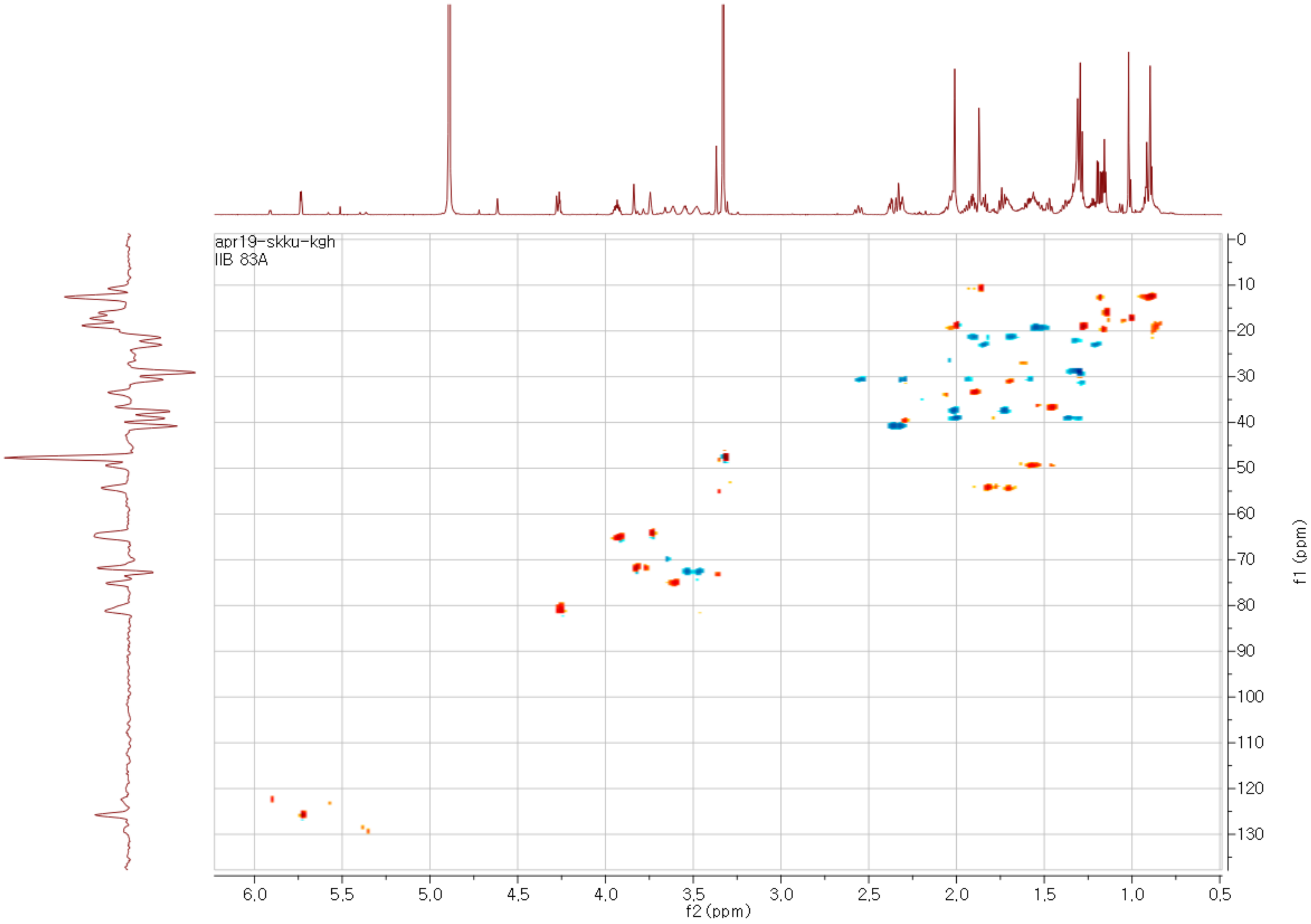

Figure S12. The HMBC spectrum of **1a** (CD_3OD)

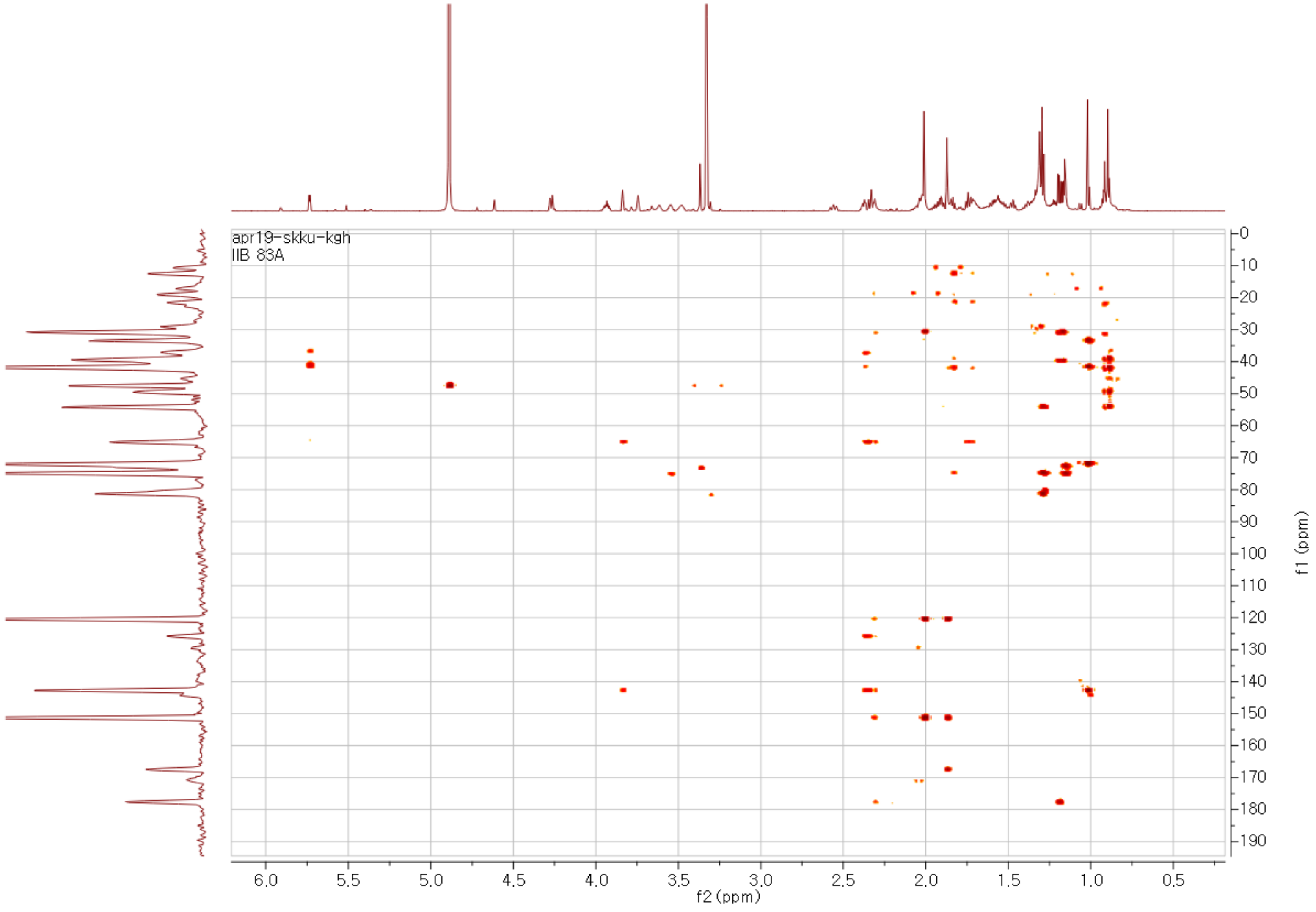

Figure S13. The ROESY spectrum of **1a** (CD_3OD)

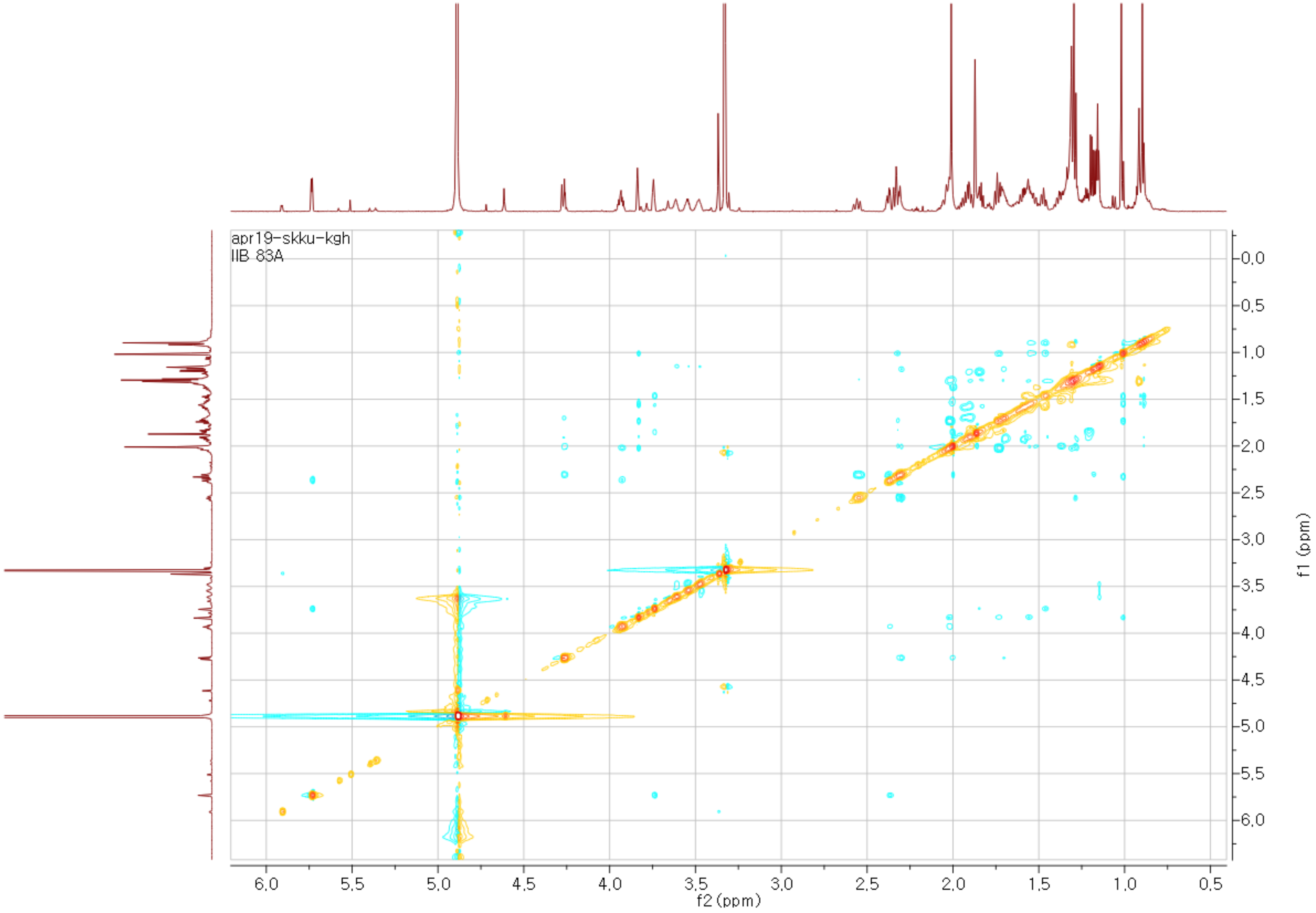

Figure S14. The ECD spectrum of **1a**

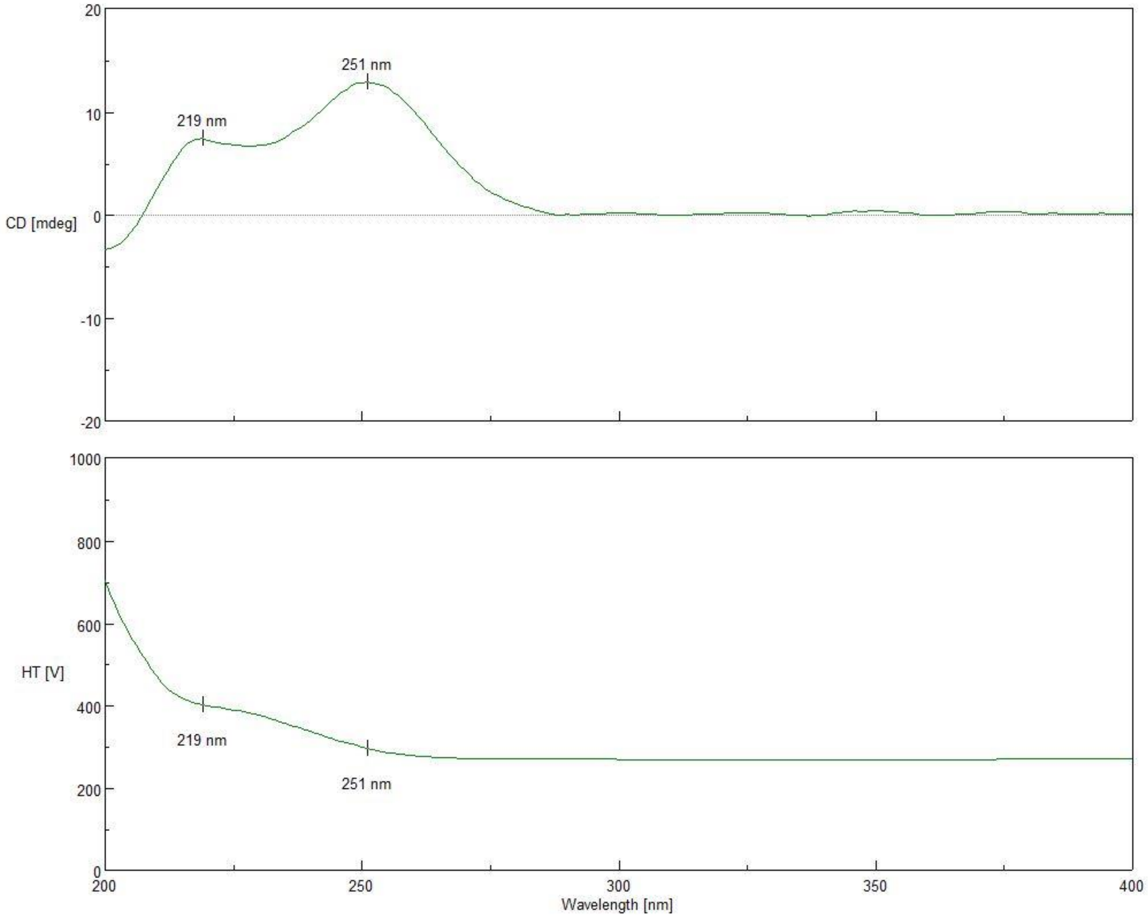

Figure S15. The HR-ESIMS data of **2**

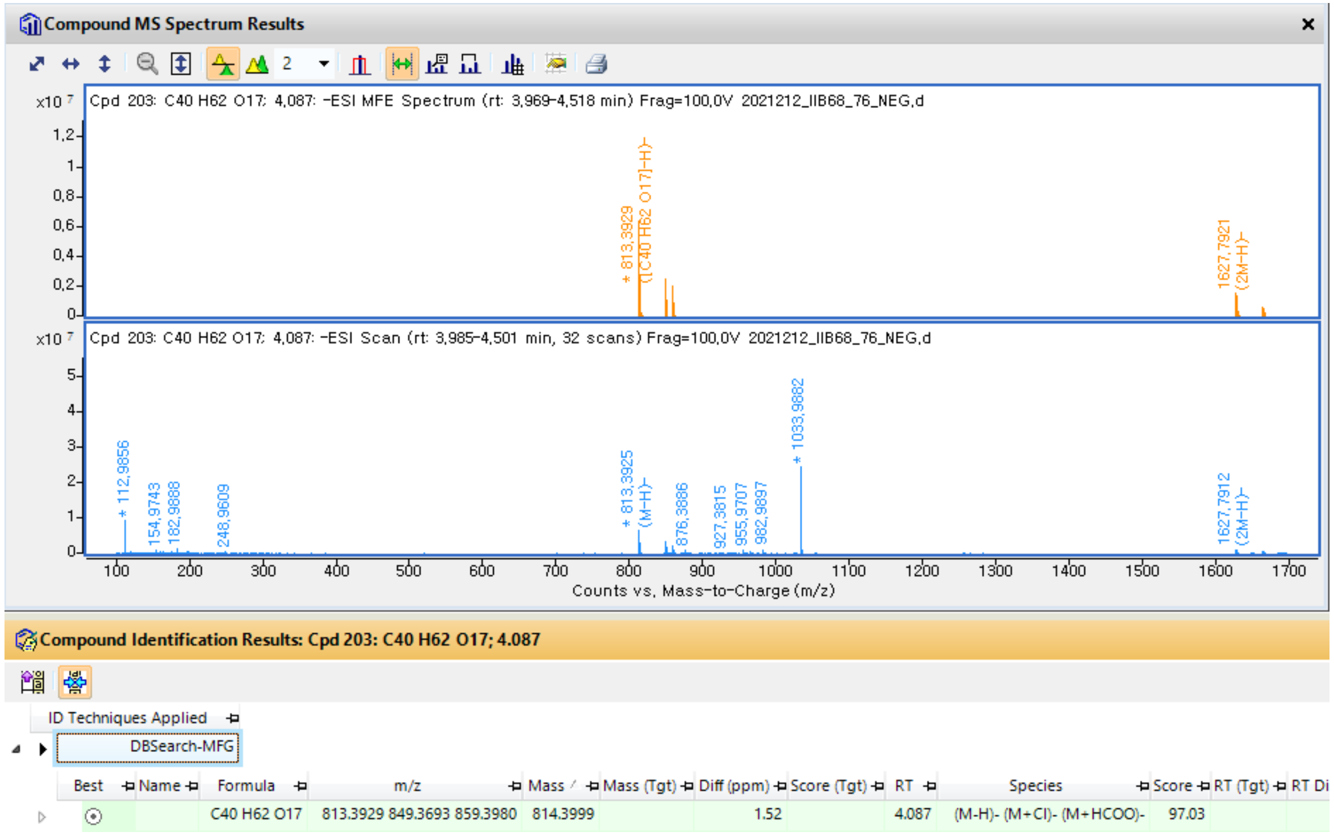

Figure S16. The ^{1}H NMR spectrum of **2** (CD_3OD, 850 MHz)

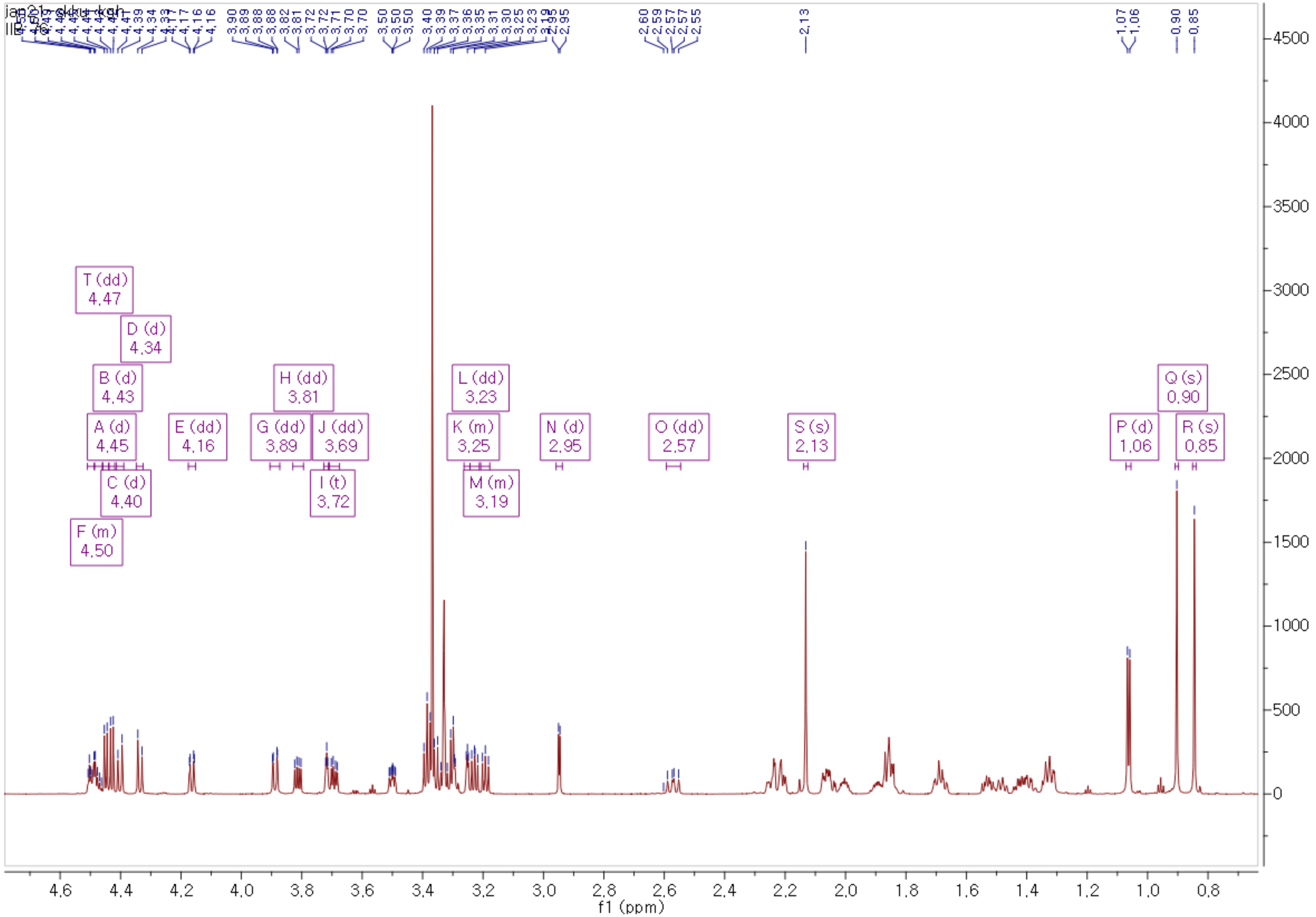

General experimental procedure

Optical rotations were measured using a JASCO P-2000 polarimeter (JASCO, Easton, MD, USA). Ultraviolet (UV) spectra were acquired on an Agilent 8453 UV-visible spectrophotometer (Agilent Technologies, Santa Clara, CA, USA). Electronic circular dichroism (ECD) spectra were measured on a JASCO J-1500 spectropolarimeter (JASCO). Infrared (IR) spectra were recorded with a Bruker IFS-66/S FT-IR spectrometer (Bruker, Karlsruhe, Germany). Nuclear magnetic resonance (NMR) spectra were recorded with a Bruker AVANCE III HD 800 NMR spectrometer with a 5 mm TCI CryoProbe operating at 850 MHz (^{1}H) and 212.5 MHz (^{13}C), with chemical shifts given in ppm (δ) for 1H and 13C NMR analyses. All HRESIMS data were obtained with a Waters Xevo G2 QTOF mass spectrometer and Synapt G2 HDMS quadrupole time-of-flight (TOF) mass spectrometer (Waters). Preparative high-performance liquid chromatography (HPLC) was performed using a Waters 1525 Binary HPLC pump with a Waters 996 Photodiode Array Detector (Waters Corporation, Milford, MA, USA) and an Agilent Eclipse C18 column (250 × 21.2 mm, 5 μm; flow rate: 5 mL/min; Agilent Technologies). Semi-preparative HPLC was performed using a Shimadzu Prominence HPLC System with SPD-20A/20AV Series Prominence HPLC UV-Vis detectors (Shimadzu, Tokyo, Japan) and a Phenomenex Luna C18 column (250 × 10 mm, 5 μm; flow rate: 2 mL/min; Phenomenex, Torrance, CA, USA). LC/MS analysis was performed on an Agilent 1200 Series HPLC system equipped with a diode array detector and 6130 Series ESI mass spectrometer using an analytical Kinetex C18 100 Å column (100 × 2.1 mm, 5 μm; flow rate: 0.3 mL/min; Phenomenex). Silica gel 60 (230-400 mesh; Merck, Darmstadt, Germany) and RP-C18 silica gel (Merck, 230-400 mesh) were used for column chromatography. The packing material for molecular sieve column chromatography was Sephadex LH-20 (Pharmacia, Uppsala, Sweden). Thin-layer chromatography (TLC) was performed with precoated silica gel F254 plates and RP-C18 F254s plates (Merck) and spots were detected under UV light or by heating after spraying with anisaldehyde-sulfuric acid.

Plant material

One-year old roots of *W. somnifera* were purchased from Seong-geo-san Farm, Cheonan, Korea in October 2016, and the plant was identified by one of the authors (K. H. Kim). A voucher specimen of the material (IDG-2016) was deposited in the herbarium of the School of Pharmacy, Sungkyunkwan University, Suwon, Korea.

Absolute configuration determination of the sugar moieties of compound 1

The aqueous layer was evaporated under a vacuum evaporator and dissolved in anhydrous pyridine (0.5 mL) with the addition of L-cysteine methyl ester hydrochloride (1.0 mg). After the reaction mixture was heated at 60 ℃ for 1 h, *o*-tolyl isothiocyanate (50 μL) was added and the

mixture was kept at 60 ℃ for 1 h. The reaction product was evaporated under a vacuum evaporator and dissolved in MeOH. After then, the dissolved reaction product was directly analyzed by LC/MS [MeOH/H_2O,1:9 → 7:3 gradient system (0–30 min), 100% MeOH (31–41 min), 0% MeOH (42–52 min); flow rate of 0.3 mL/min] using analytical Kinetex C_{18} 100 Å column (100 mm × 2.1 mm i.d., 5 µm). The sugar moieties from **1** were identified as D-glucopyranoses, based on a comparison with the retention time of an authentic sample, D-glucopyranose (t_R 18.7 min) in the LC/MS analysis.

Helicobacter pylori culture

A clinical strain of *H. pylori* 51 was provided by the *H. pylori* Korean Type Culture Collection, School of Medicine, Gyeongsang National University, Korea. The strain was grown and maintained on Brucella agar medium (BD Co., Sparks, MD, USA) supplemented with 10% horse serum (Gibco, New York, USA). The culture conditions were 37 ℃, 100% humidity, and 10% CO_2 for 2–3 days.

Anti-Helicobacter pylori activity

Twenty microliters of the bacterial colony suspension equivalent to 2×10^8–3×10^8 cfu/mL and 20 µL of test samples or controls were added to Brucella broth medium supplemented with 10% horse serum to each well in a 6-well plate. The final volume was 2 mL, and the final concentrations were 100 µg/mL and 100 µM for the fractions and compounds, respectively. After 24 h of incubation at 37 ℃, growth was assessed by measuring the optical density at 600 nm using a spectrophotometer. Quercetin and metronidazole were purchased from Sigma (St. Louis, MO, USA) and used as positive controls. The inhibition values were obtained from three independent experiments.

Anti-inflammatory activity assay

RAW 264.7 cells (6.0×10^4cells/well) were seeded into a 96-well plate and incubated overnight for adhesion. Following incubation, the cells were treated with compounds **1-4** and LPS. After 24 h incubation, supernatants were collected and treated with Griess reagent for evaluating NO concentration in the reactants. The absorbance was measured at 540 nm and NO production was calculated by referring to the nitrite standard curve.
